# Functional Imaging of the Cerebellum during Action Execution and Observation

**DOI:** 10.1093/texcom/tgab041

**Published:** 2021-06-30

**Authors:** Vassilis Raos, Helen E Savaki

**Affiliations:** Institute of Applied and Computational Mathematics, Foundation for Research and Technology—Hellas, Heraklion, Crete 70013, Greece; Department of Basic Sciences, Medical School, University of Crete, Heraklion, Crete 70013, Greece; Institute of Applied and Computational Mathematics, Foundation for Research and Technology—Hellas, Heraklion, Crete 70013, Greece; Department of Basic Sciences, Medical School, University of Crete, Heraklion, Crete 70013, Greece

**Keywords:** action execution, action observation, cerebellum, mental simulation, monkey

## Abstract

We employed the ^14^C-deoxyglucose autoradiographic method to map the activity in the cerebellar cortex of rhesus monkeys that performed forelimb movements either in the light or in the dark and of monkeys that observed forelimb movements executed by a human experimenter. The execution of forelimb movements, both in the light and in the dark, activated the forelimb representations in the cerebellar hemispheric extensions of 1) vermian lobules IV–VI and 2) vermian lobule VIIIB, ipsilaterally to the moving forelimb. Activations in the former forelimb representation involved both a paravermal and a lateral hemispheric region. Also, Crus II posterior in the ansiform lobule (the hemispheric expansion of lobule VIIB) was activated bilaterally by execution of movements in the light but not in the dark. Action observation activated the lateral-most region of the forelimb representation in the lateral hemispheric extension of vermian lobules IV–VI, as well as the crus II posterior, bilaterally. Our results demonstrate that the cerebellar cortex, in addition to its involvement in the generation of movement, is also recruited in the perception of observed movements. Moreover, our findings suggest a modularity gradient in the primate cerebellar cortex, which progresses from unimodal (medially) to multimodal (laterally) functional areas.

## Introduction

Our ability to learn fine motor skills as well as to interact socially depends on observing and interpreting the actions of other subjects. This underlines the importance of investigating where and how observed actions are represented in the primate brain. The discovery of mirror neurons in cerebral cortical areas of the macaque brain (di Pellegrino et al. 1992; Papadourakis and Raos 2017, 2019) largely contributed in this direction. Mirror neurons were found to discharge both when a monkey performs an action and when the monkey observes another individual performing the same action, and therefore their involvement in action understanding was suggested (Gallese et al. 1996; Rizzolatti et al. 1996). Also, accumulating evidence supports the existence of a “mirror system” in humans (Rizzolatti et al. 2014; Rizzolatti and Sinigaglia 2016).

In a series of high-resolution brain imaging experiments, using the quantitative ^14^C-deoxyglucose method (^14^C-DG) in nonhuman primates, we established that the cerebral cortical network of a monkey engaged in action execution with a forelimb (reaching to grasp movements) is activated by the monkey merely observing the same action (for review, see Savaki 2010). This action execution/observation network encompasses specific parieto-temporal somatosensory and temporo-occipital visual cortical regions, prefrontal and occipito-parieto-temporal association areas, as well as frontal premotor and sensorimotor cortical regions including the forelimb representation of the primary motor and somatosensory cortices (Raos et al. 2004, 2007; Evangeliou et al. 2009; Stamos et al. 2010; Kilintari et al. 2011, 2014; Raos et al. 2014; Raos and Savaki 2016, 2017). Similar effects were obtained in humans executing and observing the very same action (Simos et al. 2017). The fact that both generation and perception of an action share common neural circuits in nonhuman primates as well as in humans indicates that covert and overt actions recruit shared movement–effect representations. Therefore, we suggested that perception of an action performed by another subject triggers our previous knowledge about the act and its predicted consequences, that action perception corresponds to simulation of its overt counterpart, and that we decode the actions of others by activating our own action system, that is, by simulating the action mentally (for review, see Savaki and Raos 2019).

Although the cerebral involvement in action observation is well established in nonhuman primates as described above, the cerebellar implication is not elucidated. In humans, fMRI evidence for cerebellar recruitment during action observation is inconsistent. This is clearly demonstrated in meta-analyses of action observation studies, reporting no (Caspers et al. 2010), limited (Molenberghs et al. 2012), or robust cerebellar activations (Van Overwalle et al. 2014). However, at least 3 studies have demonstrated that the cerebellum causally contributes to the observation of others’ actions. They reported evidence that cerebellar impairments, such as cerebellar tumors (Sokolov et al. 2010), cerebellar ischemia (Cattaneo et al. 2012), or spinocerebellar ataxia (Abdelgabar et al. 2019), can affect the ability of the patients to identify others’ actions. Actually, it was reported that patients with spinocerebellar ataxia are impaired in discriminating differences in the kinematics of observed limb movements of others and therefore it was concluded that the human cerebellum is involved in perceiving movement kinematics (Abdelgabar et al. 2019). It is also established that cerebral cortical areas including the primary motor cortex (Evarts and Thach 1969; Allen and Tsukahara 1974; Lu et al. 2007), the prefrontal cortex (Kelly and Strick 2003; Bostan et al. 2013), and the posterior parietal cortex (Sasaki et al. 1977; Stein and Glickstein 1992; Clower et al. 2001; Prevosto et al. 2010) are interconnected with the cerebellum, in a closed-loop architecture, suggesting no interactions between the systems concerned with movement and cognition (Kelly and Strick 2003). Given that the cerebellum 1) encodes predictive and feedback signals not only of the effector kinematics but also of the task performance (Popa and Ebner 2018), 2) maintains information in working memory (Strick et al. 2009), 3) integrates information in both motor and nonmotor domains (Koziol et al. 2014; Sokolov et al. 2017; Popa et al. 2019), and 4) may influence cognitive and visuospatial computations in prefrontal and posterior parietal cortex (Clower et al. 2001; Middleton and Strick 2001; Ramnani 2012), its involvement in action observation could be crucial. Due to the high spatial resolution of the ^14^C-deoxyglucose method (Sokoloff et al. 1977) allowing for detection of subtle activations induced by arm-reaching/hand-grasping experiments in monkeys (Raos et al. 2007; Evangeliou et al. 2009; Raos and Savaki 2017), in the present study, we reveal the precise components of the monkey cerebellum involved in action observation. Moreover, by comparing in detail the activated cerebellar components we report here with the previously reported cerebral cortical activations induced by the same action in the same monkeys, we are able to suggest the possible cerebrocerebellar networks engaged in action observation in nonhuman primates.

## Materials and Methods

### Subjects

The cerebella from 21 adult female monkeys (Macaca mulatta) weighing between 4 and 6 kg were analyzed in the present study. Animals were purpose-bred and imported to Greece from authorized European suppliers following the appropriate rules for the intra-Union trade. Experimental protocols were approved by the Veterinary Authorities of the Region of Crete (11902/3-8-2011, 3648/18-6-2010, 989/22-2-2008, 727/13-2-2007, 512/8-2-2006, 5929 & 5930/25-11-2004, 4121/3-9-2003, 89/8-1-2002, 2621/28-8-2000, 1895/7-7-1999, 1147/7-5-1998) and complied with the national laws and the EU directives on the protection of animals used for scientific purposes. A detailed description of surgical procedures and recordings of electromyographic activity and eye position during the experimental period has been reported previously (Raos et al. 2004, 2007; Savaki et al. 2015).

In brief, the immobilization of the head during the training and experimental sessions was achieved by means of a metal bolt that was secured on mandibular plates fixed on the skull with titanium screws (Synthes). All surgical procedures were performed under general anesthesia (ketamine hydrochloride, 20 mg/kg IM, followed by sodium pentobarbital, 25 mg/kg IM) and asepsis and were followed by a recovery period of 4 weeks. Before and after surgery, antibiotics and analgesics were administered systemically. Monkeys were trained to perform their tasks continuously for at least 1 h per day for several months before the ^14^C-DG experiment, until they reached reliable performance at high success rates (>90%). Successful trials were rewarded with water delivered through a tube attached close to their mouth. On the day of the ^14^C-DG experiment, monkeys performed their tasks continuously during the entire experimental period of 45 min. The monkeys were on water controlled schedule and were never deprived of fluids. Before the initiation of training, the daily amount of consumed water was measured for each monkey. After the initiation of training, this amount of water, minus the water consumed during the training as reward, was provided to the monkeys after each training session. The animals drunk water ad libitum during weekends. Monitoring of the instantaneous position of the eye was performed either by means of the scleral search coil technique (Moschovakis et al. 2001) or with an infrared oculometer (Dr Bouis). Eye position was sampled at a rate of 500 Hz using either the Spike2 (Cambridge Electronics Design) or custom-made software. Digitized electromyograms, recorded with the use of Ag-AgCl surface electrodes from the biceps and wrist extensor muscles (gain x 2000, band-pass filter 0.3–3000 kHz), were previously reported (Raos et al. 2004).

### Behavioral Tasks

**Figure 1:**
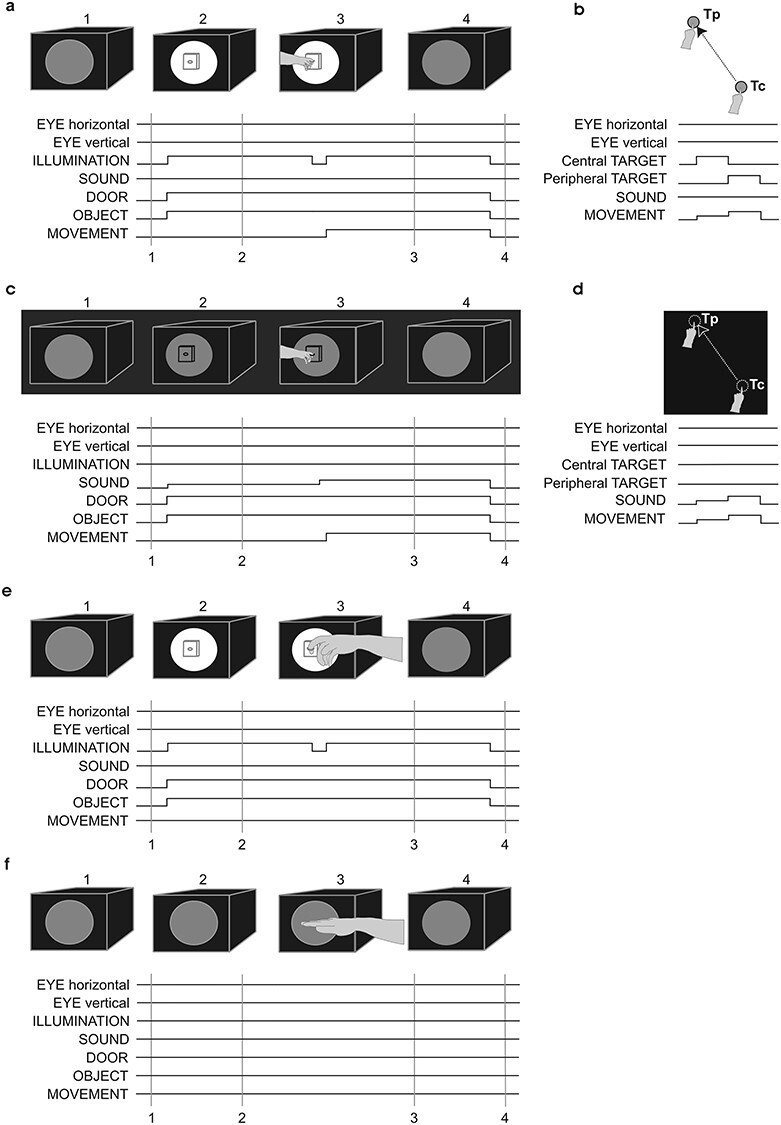
Schematics of the behavioral tasks and diagrammatic representation of the time sequence of the task events during the execution and observation conditions. (*a*) reaching-to-grasp in the light; (*b*) reaching in the light; (*c*) reaching-to-grasp in the dark; (*d*) reaching in the dark; e, observation of reaching-to-grasp movements performed by the experimenter; (*f*) observation of reaching movements performed by the experimenter. Upward deflection: on; downward deflection: off. Drawings, labeled 1–4 in panels a, c, e, and f, correspond to the times (1–4) marked with vertical lines on the diagrams of the task events.

The behavioral paradigms and the time sequence of the tasks’ events are diagrammatically presented in Figure 1. The visual stimuli, targets of the saccades and the reaching movements (see below), were red spots of 1.5° diameter, presented on a touch-screen placed 23 cm in front of the monkey. Reaching movements started from a location in the midsagittal plane at shoulder height toward a peripheral position at the up-left space (20° in amplitude and 135° in direction). A behavioral apparatus was used for the reaching-to-grasp movements. It was placed in front of the monkeys at shoulder height, 20 or 50 cm away, depending on whether the monkey or the experimenter had to execute the movement. A sliding window at the front side of the apparatus provided access to a horizontally oriented ring, which had to be grasped by either the monkey or the experimenter with the index finger inserted into it (with the hand pronated). Opening of the window resulted in illumination of the compartment thus making the object visible.

The Execution in the Light group (EL) included 3 monkeys trained to perform goal-directed forelimb movements while maintaining their gaze straight ahead. Two monkeys were trained to reach and grasp with the left forelimb, while the right forelimb was restricted (Fig. 1*a*). They were required to fixate the illuminated object behind the opened window of the grasping behavioral apparatus (window of 8° diameter around the central fixation point) for 0.7–1 s, until a dimming of the light would signal reaching, grasping, and pulling the horizontally oriented ring with the left forelimb while maintaining fixation. The movement was usually completed within 500–600 ms, while the maximum latency to reach and grasp was set at 1 s. The monkeys were allowed to move their eyes outside the window only during the intertrial intervals (ranging between 2 and 2.5 s). The third monkey was trained to perform reaching movements from a central to a peripheral visual target (20° up-left) while its gaze remained fixed on the central visual target (Fig. 1*b*). This monkey had to fixate and touch with the index of its left forelimb the central lit visual target (for 0.8–1.5 s) until illumination of the peripheral target, which signaled reaching, touching, and holding it (for 0.5–1 s) while maintaining fixation of the central target. Then the targets disappeared and the monkey was free to move its eyes and return its forelimb to the initial rest position. The monkey was allowed to perform the movement within a period ranging from 0.25 to 1.5 s after the illumination of the central target and the intertrial intervals ranged between 1 and 1.8 s.

The 5 monkeys included in the Observation (O) group were trained to maintain their gaze straight ahead while the experimenter executed arm movements in front of them. Three of the monkeys were trained to maintain gaze straight ahead while the experimenter performed reaching-to-grasp movements similar to those executed by the EL monkeys, using the same behavioral apparatus, which was located 50 cm away from the monkey (Fig. 1*e*). The experimenter was standing on the right side of the monkey and was using the right arm/hand. Both reaching and grasping components of the movement were visible to the monkey. Object and movement parameters as well as intertrial intervals and rate of responses were similar to the ones described for the EL monkeys. The other 2 monkeys were trained to maintain their gaze straight ahead while the experimenter was simply reaching toward the closed window of the apparatus (Fig. 1*f*). Therefore, these monkeys were exposed to the reaching movement of the experimenter but not to the view of hand preshaping or hand–object interaction. Both forelimbs of the O monkeys were restricted during the observation training and the ^14^C-DG experiment.

To remove the visual effect caused by plain fixation, the map of cerebellar activations induced by the Control in the Light (CL) group was subtracted from the corresponding maps of the EL and O groups of monkeys. In the CL group, we included 8 animals, which served as experimental subjects to study the representation of visuo-oculomotor space in the arcuate and prearcuate cortex (Savaki et al. 2015) and the lateral bank of the intraparietal sulcus (Savaki et al. 2010).

The specific tasks performed by the animals included in the CL group are: 2 monkeys executed horizontal saccades (1 and 2), 1 performed vertical saccades (3), 4 monkeys executed oblique upward and/or downward saccades to visual targets (4–7), and 1 fixated a central target (8). Each trial was initiated with the appearance of a central fixation target. The animals had to fixate it until it disappeared and a peripheral target was turned on signaling that a saccade to it should be executed within 1 s. Monkeys had to fixate the peripheral target for 0.3–0.8 s until it disappeared. Monkey 1 performed a full sequence of 5°, 10°, and 15° saccades to the left from the central fixation point, followed by 2 consecutive 30° saccades to the right and a 30° saccade to the left, along the horizontal meridian. Monkey 2 executed a sequence of 5°, 10°, and 15° saccades to the left, followed by a 30° saccade to the right, and then a sequence of 5°, 10°, and 15° saccades to the right, followed by a 30° saccade to the left, along the horizontal meridian. Monkey 3 performed a sequence of 5°, 10°, and 15° upward saccades from the central fixation point, followed by a 30° downward saccade, and then a sequence of 5°, 10°, and 15° downward saccades, followed by a 30° upward saccade, along the vertical meridian. Monkey 4 executed an oblique saccade 20° up-left from the central fixation point in the direction of 135°. Monkey 5 performed a sequence of 2 down-right saccades from the central fixation point 10° in amplitude and 315° in direction. Monkey 6 executed a sequence of a 20° up-left saccade (135° direction) followed by 2 down-right 10° saccades (315° direction). Monkey 7 performed a sequence of two 10° up-left saccades (135° direction) followed by one 20° down-right saccade (135° direction). Further explanation for the use of these monkeys in the control group is provided in the Results section.

The Execution in the Dark group (ED) consisted of 3 monkeys initially trained to perform goal-directed forelimb movements in the light, and subsequently trained to perform the same movements in complete darkness. Two monkeys were trained to reach and grasp with the left forelimb in complete darkness, while the right forelimb was restricted (Fig. 1*c*). A speaker was placed 25 cm in front of the monkey, in the median sagittal plane, below the behavioral apparatus. Following an auditory cue (90 Hz), each monkey had to look straight ahead toward the memorized location of the object for 0.7–1 s, until a second auditory cue (180 Hz) signaled the generation of the learned action (reaching, grasping, and pulling the memorized ring with the left forelimb) while maintaining its gaze straight ahead. The maximum latency to grasp the object was set to 1 s, although the movement was usually completed within 500–600 ms. Monkeys were allowed to move their eyes outside the window only during the intertrial intervals (ranging between 2 and 2.5 s). The third monkey performed acoustically triggered forelimb-reaching movements from a memorized central to a memorized peripheral location (20° up-left) in complete darkness while its eyes maintained a straight ahead direction (Fig. 1*d*). A speaker was placed 23 cm in front of the monkey, in the median sagittal plane, on top of the screen. Following an auditory low-frequency tone (90 Hz), the monkey had to look straight ahead toward a memorized location corresponding to the central position, to reach (within 3 s) and touch with 2 fingers (index and middle) of its left forelimb the screen at this central position (holding period 0.6–1 s). Then a high-frequency tone (180 Hz) signaled a reaching movement (within 2 s) to the memorized peripheral position (holding period 0.5–1 s), while the eyes maintained the straight-ahead direction. Intertrial intervals were 0.5–0.9 s long. To achieve complete darkness, the primate chair was enclosed within black curtains together with the behavioral apparatus, and an extra black drape was positioned in front of the monkey’s eyes.

To reveal the effects induced by reaching/grasping in the dark, the metabolic maps of the cerebellar areas from the 3 ED monkeys were compared with those obtained from the 2 Control in the Dark monkeys (CD). The CD monkeys had to remain still while listening to auditory stimuli of the same frequencies and the same sequence as the ED monkeys. Reward was delivered at random intervals to prevent association of the auditory stimuli with the reward expectancy. The total number of rewards that the CD monkeys received matched that of the ED monkeys.

### ^14^C-DG Experiment

The ^14^C-DG method is the only imaging approach to offer direct assessment of brain activity and quantitative measurement of glucose consumption (functional activity). Details of the ^14^C-DG experiment and the brain processing for autoradiography were previously described (Savaki et al. 1993; Dalezios et al. 1996; Savaki et al. 1997). On the experimental day, monkeys were subjected to femoral vein and artery catheterization under anesthesia (ketamine hydrochloride, 20 mg/kg IM). To avoid anesthesia effects on LCGU values, a recovery period of 5 h was allowed before the initiation of the ^14^C-DG experiment. Plasma glucose concentration, blood pressure, and hematocrit were measured to be within normal range. A pulse of 100 μCi/kg of 2-deoxy-D-[1-^14^C] glucose (specific activity 55 mCi/mmol, ARC) was delivered intravenously, 5 min after the monkeys started performing their tasks. Arterial samples were collected during the subsequent 45 min according to a predefined schedule to measure plasma ^14^C-DG and glucose concentrations. At 45 min, monkeys were killed by intravenous injection of 50 mg sodium thiopental in 5 mL saline followed by a saturated potassium chloride solution. The cerebella were frozen in isopentane at −50 °C and stored at −80 °C. About 1000 serial coronal sections (20 μm thick) were cut in each cerebellum, in a cryostat at −20 °C. Sections along with precalibrated ^14^C-standards were exposed to medical X-ray film (Kodak Biomax MR) to prepare autoradiographs. Quantitative densitometric analysis of autoradiographs was performed using the MCID computerized image processing system (MCID, Imaging Research, Ontario, Canada). Local cerebral glucose utilization (LCGU) values (in μmol/100 g/min) were calculated using the original operational equation of the ^14^C-DG method (Sokoloff et al. 1977) and the kinetic constants for the monkey (Kennedy et al. 1978).

### Reconstruction of Quantitative 2-Dimensional (2D) Maps

**Figure 2:**
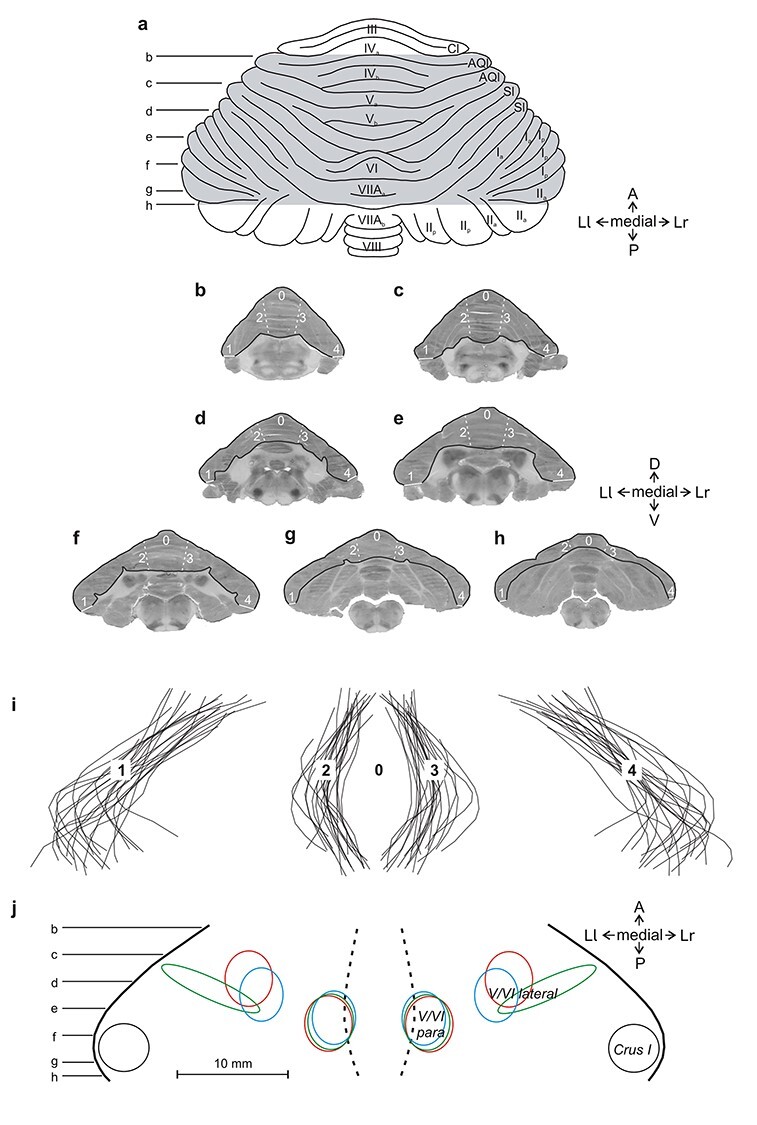
Reconstruction of 2D maps of the cerebellar cortex in the vermian lobules IV-VII and their hemispheric extensions. (*a*) Drawing of the dorsal surface of cerebellum modified from Madigan and Carpenter (Madigan and Carpenter 1971). Shaded area indicates the reconstructed cerebellar cortex. A, anterior; AQl, anterior quadrangular lobule; Cl, central lobule; Ia, Ip, IIa, IIp, Crus portions of the ansiform lobule; III-VIII, folia of the cerebellar vermis; Ll, lateral left; Lr lateral right; P, posterior; Sl, simple lobule. Horizontal lines b–h indicate the 7 different anteroposterior levels corresponding to the following 7 autoradiographic sections (*b*–*h*). The outlined gray matter in each section indicates the cortical field that was reconstructed. Zero represents the point of alignment of adjacent coronal sections in each cerebellum. Dashed white lines labeled by numbers 2 and 3 correspond to the left and right limits of the vermis, respectively. Solid white lines in each section, labeled with the numbers 1 and 4, represent the left and right edges of the reconstructed area. (*i*) Outlines of the reconstructed cortical maps drawn from all cerebella before geometrical normalization, aligned in the middle of the vermis (point 0). Black lines labeled by numbers 2 and 3 represent the limits of the vermis (left and right, respectively), whereas those labeled by numbers 1 and 4 represent the edges of the reconstructed area (left and right, respectively). (*j*) Reference map of the landmarks generated after geometrical normalization. Horizontal lines b–h indicate the anteroposterior location of the sections illustrated above. Circles and ellipses mark the location of maximum activations in the geometrically normalized map of each animal providing the values reported in the Tables 1–3. Red, green, and blue shapes correspond to the location of pixels sampled from EL, O, and ED groups, respectively. Black shape denotes that the location of maximum activation was the same in all groups.

**Figure 3:**
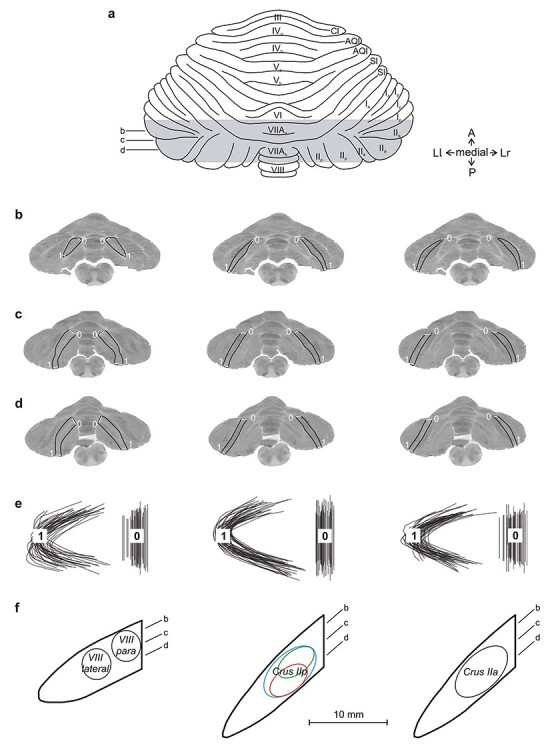
Reconstruction of 2D maps of the cerebellar cortex in the hemispheric extension of cerebellar vermian lobules. Left column: VIIIB; middle column: VIIB (Crus IIp); right column: VIIA (Crus IIa). (*a*) Drawing of the dorsal surface of cerebellum modified from Madigan and Carpenter (Madigan and Carpenter 1971). Shaded area indicates the reconstructed cerebellar cortex. Horizontal lines b–d indicate the 3 different anteroposterior levels of sectioning corresponding to the following 3 coronal sections (*b*–*d*). The gray matter with the black outline in each section indicates the cortical field that was reconstructed. Zero represents the point of alignment of adjacent sections in each cerebellum. Solid white lines in each section (0, 1) represent the mediodorsal and the ventrolateral edges of the reconstructed area, respectively. (*e*) Outlines of the reconstructed cortical maps drawn from all cerebella before geometrical normalization, aligned at the center of the reconstruction. Outlines from the right side are reflected to match those from the left. Black lines labeled with the numbers 0 and 1 represent the mediodorsal and the ventrolateral edges of the reconstructed area, respectively. (*f*) Reference map of the landmarks generated after geometrical normalization. Slanted lines b–d indicate the anteroposterior location of the sections illustrated in the corresponding panels above. Circles and ellipses mark the location of the pixels sampled from the geometrically normalized map of each animal to obtain the values for the maximum activations reported in the Tables 1–3. Red, green, and blue shapes correspond to the location of pixels sampled from EL, O, and ED groups, respectively. Black shape denotes that the location of pixels sampled for an area was the same in all groups.

We reconstructed 2D quantitative maps of the spatiointensive pattern of metabolic activity (LCGU values in μmol/100 g/min) in the mediolateral and the dorsoventral extent of the regions of interest in each cerebellum of each monkey, using the autoradiographs of the 20 μm thick coronal sections. To construct the functional 2D maps of the vermian lobules IV–VI and their hemispheric extension, a data array was generated by sampling the LCGU values along a line running from the left lateral-most edge of the section to the vermis to the right lateral most edge of the section, parallel to the surface of the cerebellum, with thickness equal to that of the cerebellar gray matter (Fig. 2). Averaging across the thickness of the gray matter may include sampling of 2 adjacent lobules that coexist within a coronal section. However, due to the wide extension of the body representations along both the anteroposterior and mediolateral dimensions, the possible sampling contamination is not expected to significantly affect the extent and intensity of activations. A distortion is expected at the anterior-most levels of these maps, because of the increased curvature of the cerebellum. In this case, sampling includes folia of more than 2 adjacent lobules and this may result in an underestimation of activations. For the functional 2-D reconstructions of 1) the hemispheric extensions of the vermian pyramidal lobule VIIIΒ, and of the parts; 2) Crus IIa, extension of vermian lobule VIIΑ; and 3) Crus IIp, extension of vermian lobule VIIΒ, of the ansiform lobule, a data array was generated by sampling the LCGU values along a line running from the mediodorsal to the ventrolateral edge of each folium, with thickness equal to that of the short axis of the folium (Fig. 3). Data arrays from every 5 adjacent autoradiographic sections were averaged (to avoid cutting artifacts) and plotted to produce one line in the 2D maps of activity. The so generated lines were aligned around a fixed point of alignment to create a map containing the cerebellar field of interest with labeled surface landmarks. The anteroposterior and the mediolateral plotting resolution of our reconstructed 2D maps equals 100 μm.

### Geometrical Normalization of the 2D Functional Maps

Before geometrical normalization, the variability in size of the different cerebella was in average ± standard deviation: anteroposterior 2.0 ± 0.3 and mediolateral 4.1 ± 0.2 cm. To facilitate comparison of the reconstructed 2D maps obtained from different animals despite subject variability, the individual 2D maps were processed to match a reference map. For the reference map of the vermian lobules IV to VI and their hemispheric extension, the section by section distances between 1) the left lateral-most tip of the section and the left vermian border, 2) the latter and the right vermian border, and 3) the latter and the right lateral-most tip of the section were measured. The average of each one of these measures was separately computed from all 21 cerebella to produce a reference map of landmarks (Fig. 2*i*). The average areal surface in mm^2^ ± SE was 272.1 ± 18.3 and 279.8 ± 18.7 for the left and the right hemispheres, respectively, and 110.6 ± 5.7 for the vermis. Subsequently, each individual LCGU map with its own landmarks was linearly transformed in MATLAB (MathWorks) to match the reference map of landmarks. The reference maps for the hemispheric extension of the vermian pyramidal lobule VIIIΒ, as well as the Crus IIp and Crus IIa of the ansiform lobule, (extensions of vermian lobules VIIA and VIIB, respectively) were similarly generated. The section-by-section distances between the dorsomedial and the ventrolateral edges of the corresponding folium were used to produce each of the reference maps of landmarks (Fig. 3*e*). The average areal surface in mm^2^ ± SE was 71.9 ± 1.8 (left: 70.8 ± 2.5, right: 73.1 ± 2.7) for the hemispheric extension of the vermian lobule VIIIΒ, 77.5 ± 2.9 (left: 75.6 ± 3.7, right: 79.5 ± 4.5) for Crus IIp, and 64 ± 2.5 (left: 61.4 ± 3.1, right: 66.6 ± 3.8) for Crus IIa. To obtain average metabolic maps within each experimental group, the LCGU value found in a certain pixel in one of the geometrically normalized 2D maps of the group was added to the value found in the pixel occupying the same position in all other maps of the same group, and the result was divided by the number of maps used. Before averaging, metabolic activity was normalized by multiplying LCGU values with a factor that was separately determined for each cerebellum. This factor equals the ratio of the mean LCGU value in unaffected areas (such as the extension of vermian lobule IX) of the cerebellum in question over the mean LCGU value obtained from the same areas after pooling all cerebella from all monkeys (Savaki et al. 1993; Gregoriou and Savaki 2003; Picard and Strick 2003). To generate a percentage difference map, the LCGU value found in a certain pixel of a geometrically normalized averaged 2D map of a control condition was subtracted from the value found in the pixel occupying the same position in a similar average map of an experimental condition, and their difference was expressed in %LCGU values using the formula (experimental − control)/control × 100. Also, to generate maps summarizing the effects of all 3 conditions, we coded in a different color (red for EL, green for O, and blue for ED) the values of the percentage difference maps exceeding 10% and then, we superimposed them in single maps. Overlap of effects induced by action execution in the light and action observation appear yellow (red + green), and overlaps between execution in the light and in the dark are in violet (red + blue). White marks the regions activated in all conditions (red + green + blue).

### Statistical Analysis

The individual geometrically normalized 2D maps were used for measurement of the normalized LCGU values in each one of the cerebellar areas mentioned in the Tables 1–3. The location of the pixels sampled for each area is illustrated in Figures 2*j* and 3*f*. The location of the pixels sampled for an area could differ among the experimental groups, depending on the location of maximum effect. In these cases, the value for the homologous area in the corresponding control was the average of the values measured from the locations used in the experimental conditions. Percent LCGU differences between experimental and control subjects in each one of these cerebellar areas were calculated as (experimental − control)/control × 100 and side-to-side differences as (left–right)/right × 100. Values in bold in the Tables 1–3 indicate statistically significant differences revealed by a mixed-model 2-way analysis of variance (ANOVA) with condition as a between-subject variable and side as a within-subject variable, followed by Tukey’s HSD post hoc test for unequal N (*P* < 0.05). The details of the statistical analyses (*F*-test values, degrees of freedom, and exact *P* values) for each of the variables and their interaction are available in Tables 4 and 5.

**Table 1:** Metabolic effects in the cerebellar cortex of monkeys induced by action execution in the light

Cerebellar cortical area	CLi	CLc	CLi/CLc	ELi	ELc	ELi/ELc	ELi/CLi	ELc/CLc
	LCGU ± SD	LCGU ± SD	%	LCGU ± SD	LCGU ± SD	%	%	%
Lobules V/VI paravermal	38.4 ± 2.6	38.4 ± 2.8	0.0	48.9 ± 3.4	40.3 ± 0.3	**21.3**	**27.3**	4.9
				*49.3* ± 4.7	*40.5* ± 0.2	*21.7*	*28.4*	*5.5*
Lobules V/VI lateral	35.4 ± 1.7	34.9 ± 1.9	1.4	45.0 ± 0.9	38.6 ± 0.4	**16.6**	**27.1**	10.6
hemisphere				*45.5 ± 0.3*	*38.4 ± 0.3*	*18.5*	*28.5*	*10.0*
Crus I	36.1 ± 6.3	35.6 ± 6.4	1.4	34.6 ± 2.9	33.2 ± 4.0	4.2	−4.2	−6.7
Lobule VIIIΒ paravermal	38.3 ± 3.6	37.4 ± 4.6	2.4	50.6 ± 1.9	37.5 ± 2.5	**34.9**	**32.1**	0.3
				*50.3 ± 2.5*	*38.4 ± 2.9*	*31.0*	*31.3*	*2.7*
Lobule VIIIΒ lateral	34.7 ± 4.0	34.8 ± 5.1	−0.3	47.3 ± 2.5	36.1 ± 1.8	**31.0**	**36.3**	3.7
hemisphere				*47.5 ± 3.5*	*36.0 ± 2.6*	*31.9*	*36.9*	*3.4*
Crus IIa	39.2 ± 5.7	38.1 ± 6.9	2.9	39.4 ± 3.6	37.7 ± 2.0	4.5	0.5	−1.1
Crus IIp	40.8 ± 3.3	40.0 ± 1.0	2.0	48.6 ± 2.2	46.0 ± 0.9	5.7	**19.1**	**15.0**
				*48.2 ± 2.9*	*45.9 ± 1.2*	*5.0*	*18.1*	*14.8*

Notes: Normalized Local Cerebral Glucose Utilization (LCGU) values (mean ± SD in μmol per 100 g per min) in the cerebellar cortex of monkeys executing forelimb movements in the light. CL values represent the average LCGU values from the cerebella of the 8 animals included in the Control in the Light group. CLi and CLc stands for values in ipsi- and contralateral cerebellar areas. ELi and ELc represent the average values of ipsi- and contralateral areas from the entire group of animals executing forelimb movements in the light (numbers in upper lines of cells), that is, from reaching and reaching-to-grasp monkeys. ELi/CLi and ELc/CLc values represent percent differences from the control, estimated with the formula *experimental* − *control)/control × 100*. For areas of the entire group (EL) displaying significant difference from the control (numbers in bold), we also provide the average values from only the 2 monkeys executing reaching-to-grasp movements (numbers in italics, lower lines of cells)

**Table 2:** Metabolic effects in the cerebellar cortex of monkeys induced by action execution in the dark

Cortical area	CDi	CDc	CDi/CDc	EDi	EDc	EDi/EDc	EDi/CDi	EDc/CDc
	LCGU ± SD	LCGU ± SD	%	LCGU ± SD	LCGU ± SD	%	%	%
Lobules V/VI paravermal	37.4 ± 1.1	38.6 ± 0.2	−3.1	49.0 ± 1.0	40.1 ± 1.1	**22.2**	**31.0**	3.9
				*49.1 ± 1.5*	*39.9 ± 1.5*	*23.1*	*31.3*	*3.4*
Lobules V/VI lateral	35.2 ± 0.1	35.6 ± 0.1	−1.1	42.7 ± 0.9	34.7 ± 1.4	**23.1**	**21.3**	−2.5
hemisphere				*43.2 ± 0.2*	*34.6 ± 1.9*	*24.9*	*22.7*	*−2.8*
Crus I	36.2 ± 1.5	34.5 ± 2.0	4.9	36.1 ± 4.2	34.8 ± 1.9	3.7	−0.3	0.9
Lobule VIIIΒ paravermal	35.5 ± 0.8	35.0 ± 0.5	1.4	48.8 ± 4.1	37.5 ± 4.6	**30.1**	**37.5**	7.1
				*50.8 ± 2.8*	*39.7 ± 3.4*	*28.0*	*43.1*	*13.4*
Lobule VIIIΒ lateral	33.8 ± 3.3	33.1 ± 3.4	2.1	43.1 ± 2.8	31.9 ± 0.8	**35.1**	**27.5**	−3.6
hemisphere				*43.7 ± 3.6*	*32.0 ± 1.1*	*36.6*	*29.3*	*−3.3*
Crus IIa	35.9 ± 1.7	33.9 ± 0.5	5.9	35.6 ± 3.6	33.2 ± 2.7	7.2	−0.8	−2.1
Crus IIp	41.1 ± 5.9	40.5 ± 5.8	1.5	38.4 ± 4.3	36.4 ± 2.9	5.5	−6.6	−10.1

Notes: Normalized Local Cerebral Glucose Utilization (LCGU) values (mean ± SD in μmol per 100 g per min) in the cerebellar cortex of monkeys executing forelimb movements in the dark. CDi, CDc values represent the average LCGU values from the cerebella of the 2 Control in the Dark monkeys (ipsi- and contralateral cerebellar areas, respectively). EDi and EDc values represent the average from the ipsi- and contralateral parts of the cerebella of the 3 animals executing forelimb movements (reaching and reaching-to-grasp) in the dark. EDi/CDi and EDc/CDc values represent percent differences from the control, estimated with the formula *experimental* − *control)/control × 100*. Average values from the entire ED group including both reaching and reaching-to-grasp monkeys are provided in the upper lines of cells. Also, for areas displaying significant difference from the control (numbers in bold), the average values only from the 2 monkeys executing reaching-to-grasp movements (numbers in italics, lower lines of cells) are provided

**Table 3:** Metabolic effects in the cerebellar cortex of monkeys induced by action observation

Cerebellar cortical area	CLi	CLc	CLi/CLc	Oi	Oc	Oi/Oc	Oi/CLi	Oc/CLc
	LCGU ± SD	LCGU ± SD	%	LCGU ± SD	LCGU ± SD	%	%	%
Lobules V/VI paravermal	38.4 ± 2.6	38.4 ± 2.8	0.0	41.2 ± 1.5	40.4 ± 1.7	2.0	7.3	5.2
Lobules V/VI lateral	35.4 ± 1.7	34.9 ± 1.9	1.4	40.2 ± 0.5	39.3 ± 2.1	2.3	**13.6**	**12.6**
hemisphere				*39.5 ± 1.1*	*38.6 ± 2.1*	*2.3*	*11.6*	*10.6*
Crus I	36.1 ± 6.3	35.6 ± 6.4	1.4	37.8 ± 1.9	36.6 ± 1.5	3.3	4.7	2.8
Lobule VIIIΒ paravermal	38.3 ± 3.6	37.4 ± 4.6	2.4	36.5 ± 2.4	36.1 ± 1.9	1.1	−4.7	−3.5
Lobule VIIIΒ lateral hemisphere	34.7 ± 4.0	34.8 ± 5.1	−0.3	35.9 ± 1.7	34.5 ± 1.5	4.1	3.5	−0.9
Crus IIa	39.2 ± 5.7	38.1 ± 6.9	2.9	37.8 ± 2.9	37.0 ± 2.5	2.2	−3.6	−2.9
Crus IIp	40.8 ± 3.3	40.0 ± 1.0	2.0	46.2 ± 2.0	47.0 ± 2.3	−1.7	**13.2**	**17.5**
				*45.6 ± 2.6*	*45.4 ± 0.4*	*0.4*	*11.8*	*13.5*

Normalized Local Cerebral Glucose Utilization (LCGU) values (mean ± SD in μmol per 100 g per min) in the cerebellar cortex of monkeys observing forelimb movements in the light. CL values represent the average LCGU values from the cerebella of the 8 animals included in the Control in the Light group. Ipsi- and contralateral parts of the cerebella are represented by i and c, respectively. O values represent the average from the cerebella of the 5 animals observing forelimb movements, that is, from both reaching and reaching-to-grasp monkeys (numbers in upper lines of cells). For the areas displaying statistically significant differences from the control (bold values), we also provide separately the average values from the 3 animals observing reaching-to-grasp movements (numbers in italics, lower lines of cells)

## Results

**Figure 4:**
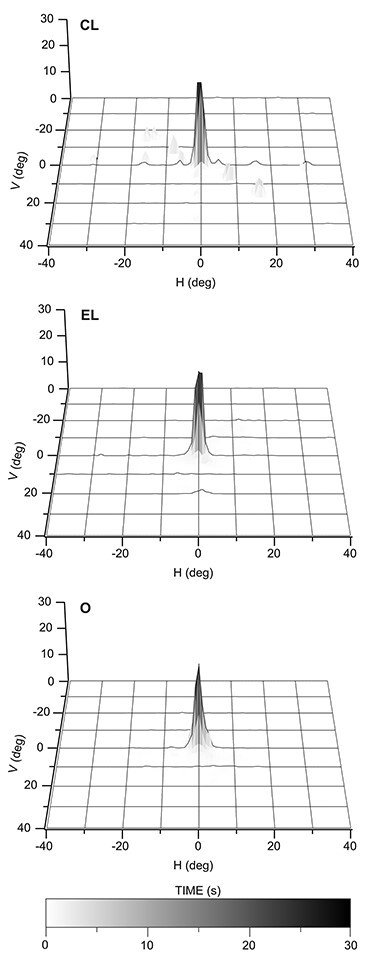
Three-dimensional histograms of the dwell time of the line of sight as a function of eye position during the 10 first minutes of the ^14^C-DG experiment. (CL) Averaged oculomotor behavior from the 8 monkeys of the CL group. (EL) Averaged oculomotor behavior from the 3 monkeys executing forelimb movements in the light. (O) Averaged oculomotor behavior from the 5 monkeys observing forelimb movements. Horizontal axis (H; *x*) and vertical axis (V; *y*) in degrees and *z*-axis in seconds. Gray scale bar indicates time in seconds.

**Figure 5:**
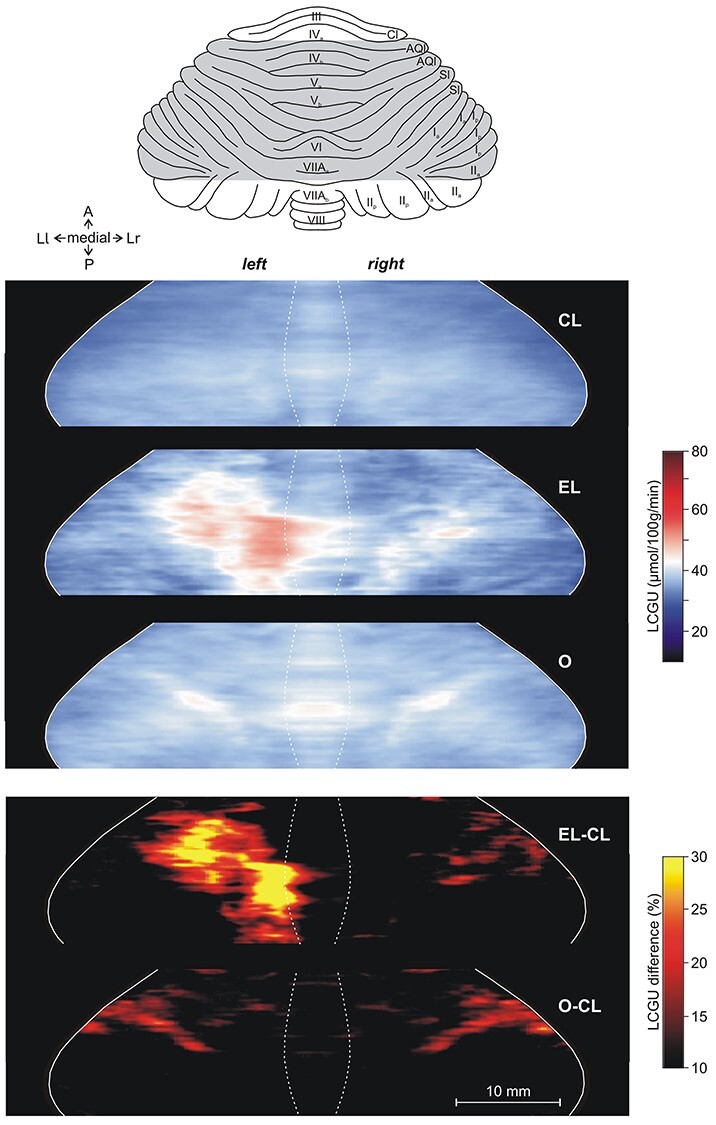
Effects induced by action execution in the light and action observation in the vermian lobules IV–VII and their hemispheric extension. Top: Drawing of the dorsal surface of cerebellum modified from Madigan and Carpenter (Madigan and Carpenter 1971). Shaded area indicates the reconstructed cerebellar cortex. CL: Quantitative 2D averaged map of metabolic activity from the cerebella of the 8 monkeys included in the Control in the Light group. Dotted lines represent the borders of vermis. EL: Quantitative 2D averaged map of metabolic activity from the cerebella of the 3 monkeys executing forelimb movements in the light. O: Quantitative 2D averaged map of metabolic activity from the cerebella of the 5 monkeys observing movements executed by the experimenter. Blue–white–red color bar indicates normalized LCGU values in μmol/100 g/min. EL-CL: Map of net effects induced by action execution in the light expressed as percentage LCGU differences from the Control in the Light [calculated as (EL-CL)/CL^*^100]. O-CL: Map of net effects induced by action observation expressed as percentage LCGU differences from the Control in the Light [calculated as (O-CL)/CL^*^100]. Black–red–yellow color bar indicates % LCGU differences from the CL. Other conventions as in Figure 2.

**Figure 6:**
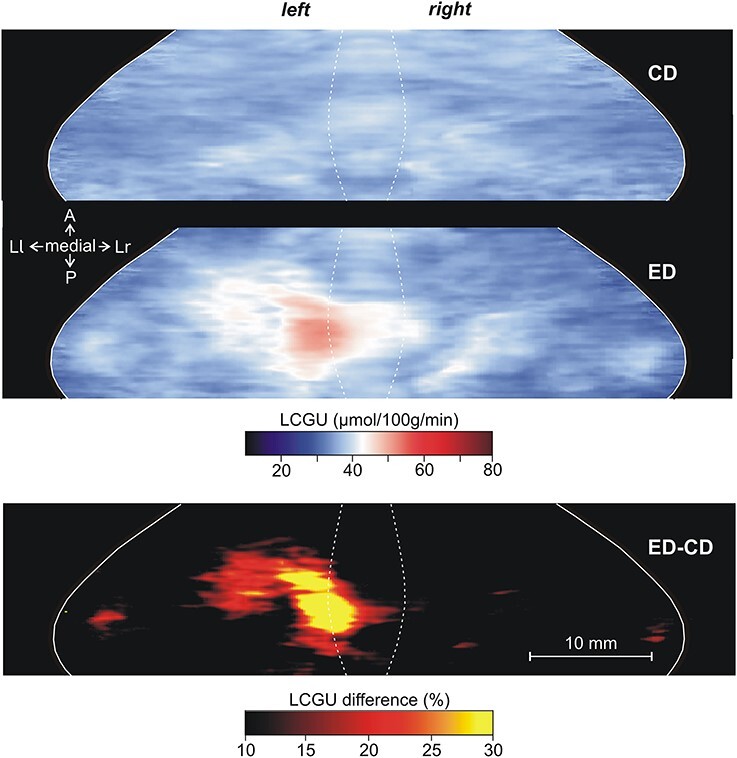
Effects induced by action execution in the dark in the vermian lobules IV-VII and their hemispheric extension. CD: Quantitative 2D averaged map of metabolic activity from the cerebella of the 2 monkeys included in the Control in the Dark group. ED: Quantitative 2D averaged map of metabolic activity from the cerebella of the 3 monkeys executing forelimb movements in the dark. Blue–white–red color bar indicates normalized LCGU values in μmol/100 g/min. ED-CD: Map of net effects induced by action execution in the dark expressed as percentage LCGU differences from the Control in the dark [calculated as (ED-CD)/CD^*^100]. Black–red–yellow color bar indicates % LCGU differences from the CD. Other conventions as in Figure 5.

**Figure 7:**
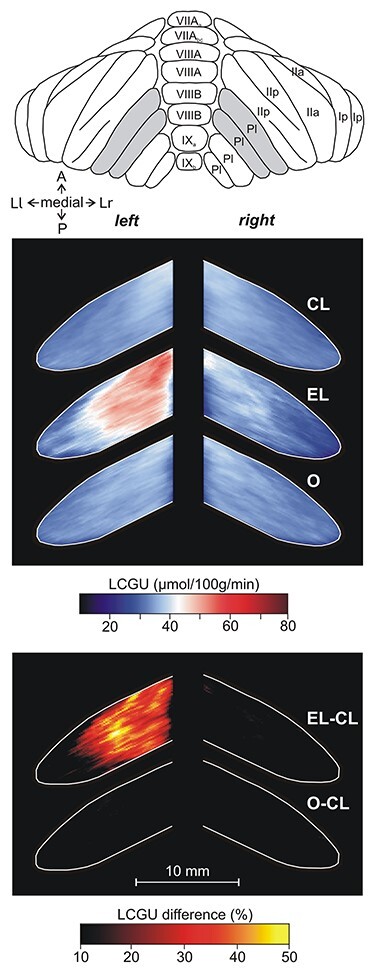
Effects induced by action execution in the light and action observation in the hemispheric extension of the vermian pyramidal lobule VIIIB. Top: Drawing of the posterior surface of cerebellum modified from Madigan and Carpenter (Madigan and Carpenter 1971). Shaded area indicates the reconstructed cerebellar cortex. A, anterior; Ia, Ip, IIa, IIp, Crus portions of the ansiform lobule; Ll, lateral left; Lr lateral right; P, posterior; Pl, paramedian lobule; VII-IX, folia of the cerebellar vermis. CL: Quantitative 2D averaged map of metabolic activity from the cerebella of the 8 monkeys included in the Control in the Light group. EL: Quantitative 2D averaged map of metabolic activity from the cerebella of the 3 monkeys executing forelimb movements in the light. O: Quantitative 2D averaged map of metabolic activity from the cerebella of the 5 monkeys observing movements executed by the experimenter. Blue–white–red color bar indicates normalized LCGU values in μmol/100 g/min. EL-CL: Map of net effects induced by action execution in the light expressed as percentage LCGU differences from the Control in the Light [calculated as (EL-CL)/CL^*^100]. O-CL: Map of net effects induced by action observation expressed as percentage LCGU differences from the Control in the Light [calculated as (O-CL)/CL^*^100]. Black–red–yellow color bar indicates % LCGU differences from the CL. Other conventions as in Figures 3 and 5.

**Figure 8:**
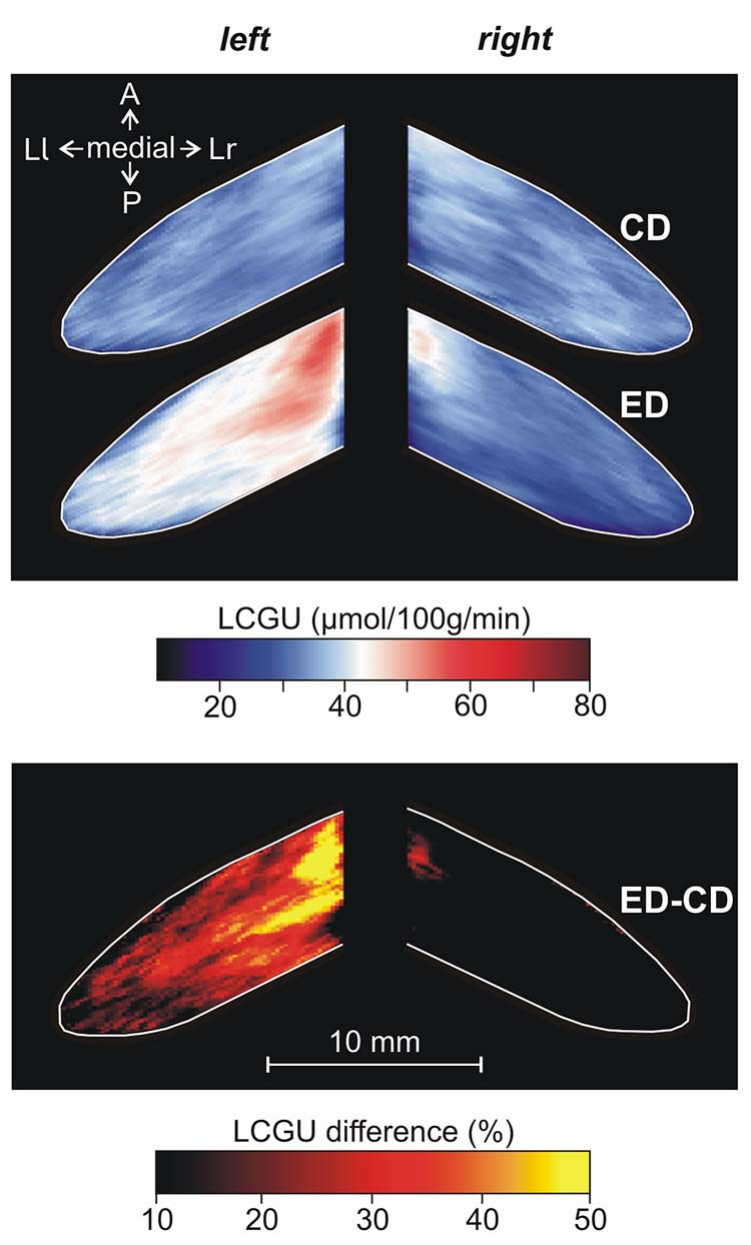
Effects induced by action execution in the dark in the hemispheric extension of the vermian pyramidal lobule VIIIB. CD: Quantitative 2D averaged map of metabolic activity from the cerebella of the 2 monkeys included in the Control in the Dark group. ED: Quantitative 2D averaged map of metabolic activity from the cerebella of the 3 monkeys executing forelimb movements in the dark. Blue–white–red color bar indicates normalized LCGU values in μmol/100 g/min. ED-CD: Map of net effects induced by action execution in the dark expressed as percentage LCGU differences from the Control in the dark [calculated as (ED-CD)/CD^*^100]. Black–red–yellow color bar indicates % LCGU differences from the CD. Other conventions as in Figure 7.

**Figure 9:**
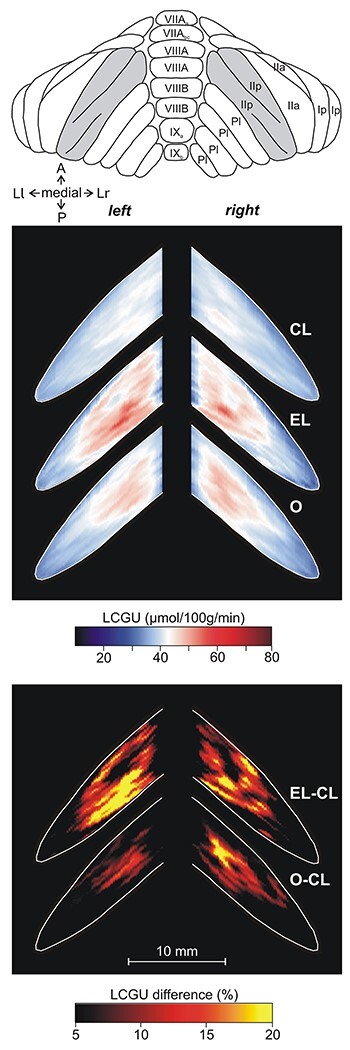
Effects induced by action execution in the light and action observation in the Crus IIp of the ansiform lobule, extension of vermian lobule VIIB. Top: Drawing of the posterior surface of cerebellum. CL: Quantitative 2D averaged map of metabolic activity from the cerebella of the 8 monkeys included in the Control in the Light group. EL: Quantitative 2D averaged map of metabolic activity from the cerebella of the 3 monkeys executing forelimb movements in the light. O: Quantitative 2D averaged map of metabolic activity from the cerebella of the 5 monkeys observing movements executed by the experimenter. Blue–white–red color bar indicates normalized LCGU values in μmol/100 g/min. EL-CL: Map of net effects induced by action execution in the light expressed as percentage LCGU differences from the Control in the Light [calculated as (EL-CL)/CL^*^100]. O-CL: Map of net effects induced by action observation expressed as percentage LCGU differences from the Control in the Light [calculated as (O-CL)/CL^*^100]. Black–red–yellow color bar indicates % LCGU differences from the CL. Other conventions as in Figures 3 and 5.

**Figure 10:**
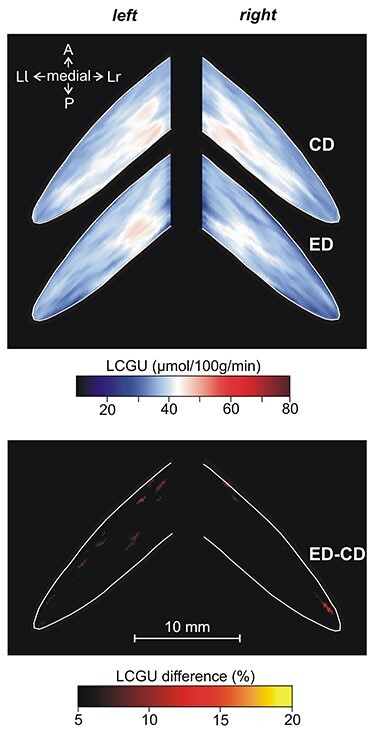
Effects induced by action execution in the dark in the Crus IIp of the ansiform lobule, extension of vermian lobule VIIB. CD: Quantitative 2D averaged map of metabolic activity from the cerebella of the 2 monkeys included in the Control in the Dark group. ED: Quantitative 2D averaged map of metabolic activity from the cerebella of the 3 monkeys executing forelimb movements in the dark. Blue–white–red color bar indicates normalized LCGU values in μmol/100 g/min. ED-CD: Map of net effects induced by action execution in the dark expressed as percentage LCGU differences from the Control in the dark [calculated as (ED-CD)/CD^*^100]. Black–red–yellow color bar indicates % LCGU differences from the CD. Other conventions as in Figure 9.

**Figure 11:**
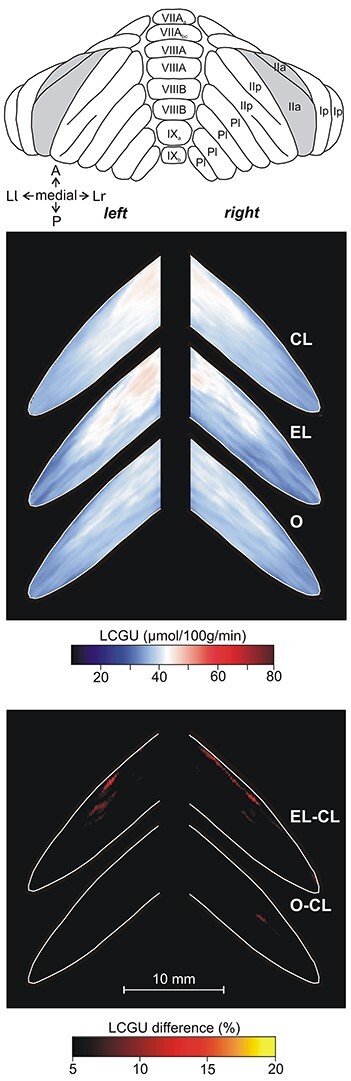
Effects induced by action execution in the light and action observation in the Crus IIa of the ansiform lobule, extension of vermian lobule VIIA. Top: Drawing of the posterior surface of cerebellum. CL: Quantitative 2D averaged map of metabolic activity from the cerebella of the 8 monkeys included in the Control in the Light group. EL: Quantitative 2D averaged map of metabolic activity from the cerebella of the 3 monkeys executing forelimb movements in the light. O: Quantitative 2D averaged map of metabolic activity from the cerebella of the 5 monkeys observing movements executed by the experimenter. Blue–white–red color bar indicates normalized LCGU values in μmol/100 g/min. EL-CL: Map of net effects induced by action execution in the light expressed as percentage LCGU differences from the Control in the Light [calculated as (EL-CL)/CL^*^100]. O-CL: Map of net effects induced by action observation expressed as percentage LCGU differences from the Control in the Light [calculated as (O-CL)/CL^*^100]. Black–red–yellow color bar indicates % LCGU differences from the CL. Other conventions as in Figures 3 and 5.

**Figure 12:**
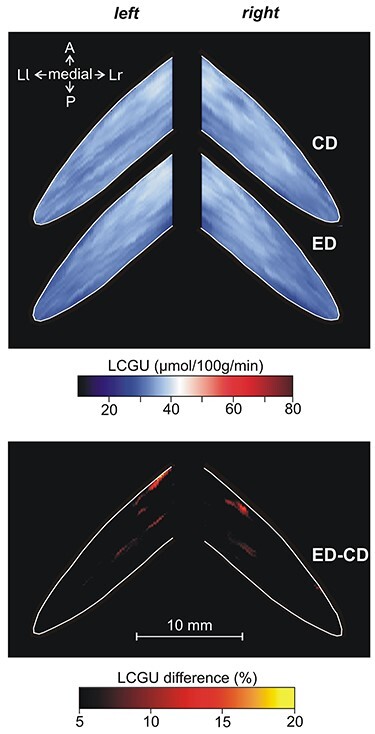
Effects induced by action execution in the dark in the Crus IIa of the ansiform lobule, extension of vermian lobule VIIA. CD: Quantitative 2D averaged map of metabolic activity from the cerebella of the 2 monkeys included in the Control in the Dark group. ED: Quantitative 2D averaged map of metabolic activity from the cerebella of the 3 monkeys executing forelimb movements in the dark. Blue–white–red color bar indicates normalized LCGU values in μmol/100 g/min. ED-CD: Map of net effects induced by action execution in the dark expressed as percentage LCGU differences from the Control in the dark [calculated as (ED-CD)/CD^*^100]. Black–red–yellow color bar indicates % LCGU differences from the CD. Other conventions as in Figure 11.

On the day of the ^14^C-DG experiment, all monkeys were engaged in their tasks for the whole duration of the experiment (45 min). The number of forelimb movements (±SD) executed or observed as well as the amount of time (±SD) the monkeys spent fixating during the ^14^C-DG experiment is reported in Tables 6 and 7. All maps displayed in Figures 4–12 correspond to 2D reconstructions of the metabolic activity in parts of the cerebellar cortex, averaged in each group. More specifically, we reconstructed 2D quantitative functional maps, which we normalized geometrically. This allowed for the generation of averaged maps per condition and for quantitative comparison of activations in homologous cerebellar areas between conditions. 

**Table 4:** Statistical details of the 3 × 2 ANOVA (condition: CL, EL, O; Side: left, right)

Cerebellar cortical area	Condition (between)			Side (within)			Interaction		
	*F* value	df	*P* value	*F* value	df	*P* value	*F* value	df	*P* value
Lobules V/VI paravermal	8.48	2, 13	0.00438994	48.74	1, 13	0.00000961	35.36	2, 13	0.00000553
Lobules V/VI lateral hemisphere	28.72	2, 13	0.00001698	31.89	1, 13	0.00007970	13.96	2, 13	0.00057966
Crus I	0.43	2, 13	0.66077185	5.11	1, 13	0.04154618	0.32	2, 13	0.73415970
Lobule VIII paravermal	5.59	2, 13	0.01774430	128.50	1, 13	0.00000004	77.81	2, 13	0.00000006
Lobule VIII lateral hemisphere	4.54	2, 13	0.03188622	89.40	1, 13	0.00000034	54.35	2, 13	0.00000048
Crus IIa	0.11	2, 13	0.89732431	6.57	1, 13	0.02358643	0.27	2, 13	0.76762986
Crus IIp	25.46	2, 12	0.00004809	1.22	1, 12	0.29099742	1.45	2, 12	0.27303627

**Table 5:** Statistical details of the 2 × 2 ANOVA (condition: CD, ED; Side: left, right)

Cerebellar cortical area	Condition (between)			Side (within)			Interaction		
	*F* value	df	*P* value	*F* value	df	*P* value	*F* value	df	*P* value
Lobules V/VI paravermal	62.18	1, 3	0.00425005	107.49	1, 3	0.00191459	181.98	1, 3	0.00088087
Lobules V/VI lateral hemisphere	42.52	1, 3	0.00732725	30.46	1, 3	0.01171531	36.25	1, 3	0.00918158
Crus I	0.001	1, 3	0.98127554	1.33	1, 3	0.33247598	0.02	1.3	0.88523327
Lobule VIII paravermal	13.60	1, 3	0.03455921	20.87	1, 3	0.01967133	17.49	1, 3	0.02492127
Lobule VIII lateral hemisphere	33.97	1, 3	0.01006121	150.13	1, 3	0.00117077	115.29	1, 3	0.00172733
Crus IIa	0.05	1, 3	0.84266409	12.73	1, 3	0.03759409	0.08	1, 3	0.79634091
Crus IIp	0.51	1, 3	0.52533767	5.70	1, 3	0.09700320	2.40	1, 3	0.21915192

**Table 6:** Number of forelimb movements (±SD) executed or observed during the ^14^C-DG experiment

Group	0–10 min	11–20 min	21–30 min	31–40 min	0–45 min
EL	98 ± 21	86 ± 18	80 ± 15	72 ± 22	371 ± 19
ED	106 ± 23	95 ± 21	94 ± 21	89 ± 25	424 ± 23
O	124 ± 18	121 ± 15	107 ± 20	94 ± 25	491 ± 20

**Table 7:** Amount of time (min ± SD) the monkeys spent fixating during the ^14^C-DG experiment

Group	0–10 min	11–20 min	21–30 min	31–40 min	0–45 min
CL	8.33 ± 1.3	8.01 ± 1.3	7.70 ± 1.3	6.58 ± 1.5	34.02 ± 1.4
EL	7.09 ± 0.5	7.00 ± 0.5	6.74 ± 0.2	6.28 ± 0.2	30.41 ± 0.4
O	7.22 ± 2.7	7.35 ± 2.2	6.78 ± 2.8	6.45 ± 2.8	31.00 ± 2.7

The EL and ED monkeys performed an average of 8 and 9 movements per min, respectively, and kept their gaze straight ahead within a window of 10° × 10° for about 70% of the time during the entire period of the ^14^C-DG experiment. The average movements of the EL and ED monkeys performed during the entire period of the ^14^C-DG experiment as well as during the consecutive 10 min intervals are shown in Table 6. Pooling together the reaching and the reaching-to-grasp monkeys in one group (EL) was indicated by the fact that the quantitative cerebellar metabolic maps of the reaching and the reaching-to-grasp monkeys were very similar. In fact, Table 1 demonstrates the values of glucose consumption in the EL group (upper line of cells) as well as the corresponding values in the reaching-to-grasp condition separately (lower line of cells). Reaching and reaching-to-grasp monkeys were also pooled together in the ED group for the same reason, and Table 2 demonstrates the effects in the entire group as well as in the reaching-to-grasp condition separately.

During the ^14^C-DG experiment, CL monkeys fixated the central fixation point and the peripheral targets for about 75% of the time. Table 7 illustrates the amount of time the monkeys spent fixating during the entire period of the ^14^C-DG experiment as well as during the consecutive 10 min intervals. Figure 4 illustrates the averaged oculomotor behavior from 1) the 8 monkeys of the CL group, 2) the 3 monkeys of the EL group, and 3) the 5 monkeys of the O group during the 10 first minutes of the ^14^C-DG experiment. In this figure, the 3D histogram of the oculomotor behavior averaged across the monkeys of the CL group shows that by averaging the effects of several animals (8 monkeys), which execute saccades to several different targets, we average out the effect of particular eye movements and we emphasize the effect of fixation. Figure 4 and Table 7 demonstrate that the monkeys of all 3 groups spent about the same amount of time fixating the target. More specifically, the time of fixation during the 10 first minutes of the ^14^C-DG experiment was in minutes ±SD: 8.33 ± 1.3 for the CL group, 7.09 ± 0.5 for the EL group, and 7.22 ± 2.7 for the O group. Apparently, the CL group is appropriate to control the effect caused by fixation. As for the saccadic effect, if any after averaging out the effect of particular eye movements as described above, the cerebellar areas known to be affected by saccades have not been included in our study. More specifically, the oculomotor cerebellum includes the flocculus and paraflocculus, the uvula, and nodulus as well as the oculomotor vermis and the fastigial and interpositus nuclei (Noda and Fujikado 1987; Fuchs et al. 1993; Ohtsuka and Noda 1995; Kojima et al. 2010; Thier and Markanday 2019), whereas we report results within the cerebellar hemispheric extensions of vermian lobules IV–VI and VIIIΒ, as well as in the cerebellar hemispheres. Indeed, a recent neuroimaging study in humans reported that the cerebellar regions activated by eye movements are located medial to those activated by action observation (Abdelgabar et al. 2019). Ιn the counterfactual scenario that an area affected by forelimb movements (in EL and O groups) would be also affected by execution of saccades (in the CL group), we would simply risk to underestimate the effects induced by forelimb movements, because in our study we subtract the control (CL) from the experimental (EL and O) activations. Finally, the gaze of the CD monkeys was distributed rather evenly in the oculomotor space, as expected.

All monkeys performing forelimb movements (EL and ED) exhibited activation of specific regions in the cerebellum as compared with their corresponding control groups (CL and CD, respectively). Explicitly, higher metabolic activity was displayed in the left (ipsilateral to the moving forelimb) than in the right cerebellar hemispheric extension of vermian lobules V and VI (i.e., in the culmen and simplex lobules, respectively) of the executing monkeys (Figs 5EL and6ED) as compared with their corresponding control groups (Figs 5CL and6CD). However, the degree of activation was not the same in the 2 activated subdivisions of the cerebellar hemispheric extension of lobules V/VI (Tables 1 and 2; Figs 5EL and6ED). These 2 subdivisions (paravermal and lateral hemispheric) could actually correspond to the 2 zones associated with the forelimb representation in the primary somatosensory–motor cortex, zones C2 and D2 in monkeys (for review, see Voogd 2014, see also discussion). To quantify the net effects induced in lobules V/VI by action execution in the light, LCGU values obtained from the control monkeys (averaged CL metabolic map) were subtracted from the corresponding values of the grasping in the light (averaged EL map) monkeys (Fig. 5EL-CL). Similarly, to estimate the quantitative net effects induced in lobules V/VI by action execution in the dark, we subtracted the averaged CD metabolic map from the averaged ED map (Fig. 6ED-CD). The latter 2 figures, displaying the net effects of action execution in the light and in the dark, demonstrate that during execution in the dark the activation in the paravermal zone of vermian lobules V/VI is higher than that in the lateral hemispheric zone, whereas during execution in the light, these 2 regions are equally activated. These activations have been measured and are presented in Tables 1 and 2.

The O monkeys observed an average of 11 movements per min and fixated within a rectangle window 10° × 10° for about 70% of the time during the entire period of the ^14^C-DG experiment. Pooling together the monkeys observing reaching with those observing reaching-to-grasp movements in one group (O) was indicated by the fact that the quantitative cerebellar metabolic maps of the 2 subgroups were very similar. In fact, Table 3 demonstrates the values of glucose consumption in the entire O group (upper lines of cells) as well as the corresponding values of only the monkeys observing reaching-to-grasp movements (lower lines of cells). Apparently, observation of the grasping component of movements, that is, 1) the object, 2) the preshaping of the hand, and 3) the hand–object interaction, did not add anything to the activation induced by the observation of simple reaching movements. To reveal the specific region activated by action observation in the hemispheric extension of vermian lobule V (culmen) and VI (simplex), we generated an averaged quantitative 2D map of metabolic activity from the monkeys observing reaching/grasping movements (O map in Fig. 5), and we compared it with the corresponding averaged control map (CL map in Fig. 5). Subtraction of CL map from the O map revealed that action observation induced significant activation only in a restricted zone of the lateral-most cerebellar extension of lobules V/VI (Table 3; Fig. 5O-CL). Interestingly, this activation for action observation was bilateral in contrast to the ipsilateral activations for action execution, which is reminiscent of the effects in the motor/premotor and parietal cerebral cortex being bilateral for observation and only contralateral to the moving forelimb for execution (Raos et al. 2004, 2007; Evangeliou et al. 2009).

As demonstrated in Tables 1–3, and illustrated in Figures 5 and 6, Crus I of the ansiform lobule, extension of vermian lobule VIIA, was not affected by any of the conditions under investigation. In other words, neither action execution in the light or in the dark nor action observation induced any effect in ansiform Crus I.

Figures 7 and 8, as well as Tables 1 and 2 demonstrate that the cerebellar biventral lobule, that is, the hemispheric extension of the vermian pyramidal lobule VIIIΒ, was activated during action execution, both in the light (Fig. 7EL) and in the dark (Fig. 8ED) as compared with the corresponding control group (Figs 7CL and8CD), in the side ipsilateral to the moving forelimb. Similar to the case of lobules V/VI above, the activation in the paravermal region of lobule VIIIΒ was stronger for execution in the dark (ED-CD), whereas the activation in more lateral hemispheric extension, which receives more visual projections (Evarts and Thach 1969; Glickstein et al. 1994), was stronger for execution in the light (EL-CL). Table 3 and Figure 7O and O-CL demonstrate that action observation induced no effect in the cerebellar biventral lobule VIIIΒ.

Tables 1 and 2 as well as Figures 9 and 10 demonstrate that Crus IIp of the ansiform lobule, extension of vermian lobule VIIB, was activated bilaterally by execution in the light (Fig. 9EL and EL-CL) but remained unaffected by action execution in the dark (Fig. 10ED and ED-CD). Interestingly, Crus IIp, which receives input from premotor and parietal cortical areas and is considered to be a cognitive area (Hashimoto et al. 2010; Prevosto et al. 2010; Voogd 2014; Schmahmann 2019; Schmahmann et al. 2019), was also bilaterally activated by action observation in our study (Fig. 9O and O-CL). In contrast, Crus IIa of ansiform lobule, extension of vermian lobule VIIA, which is predominantly interconnected with the prefrontal cerebral cortical area 46 (Bostan et al. 2013), was not affected by any of the conditions we studied. In other words, neither action execution in the light or in the dark nor action observation induced any effect in ansiform Crus IIa, (see Tables 1–3; Figs 11EL-CL, O-CL and12ED-CD).

**Figure 13:**
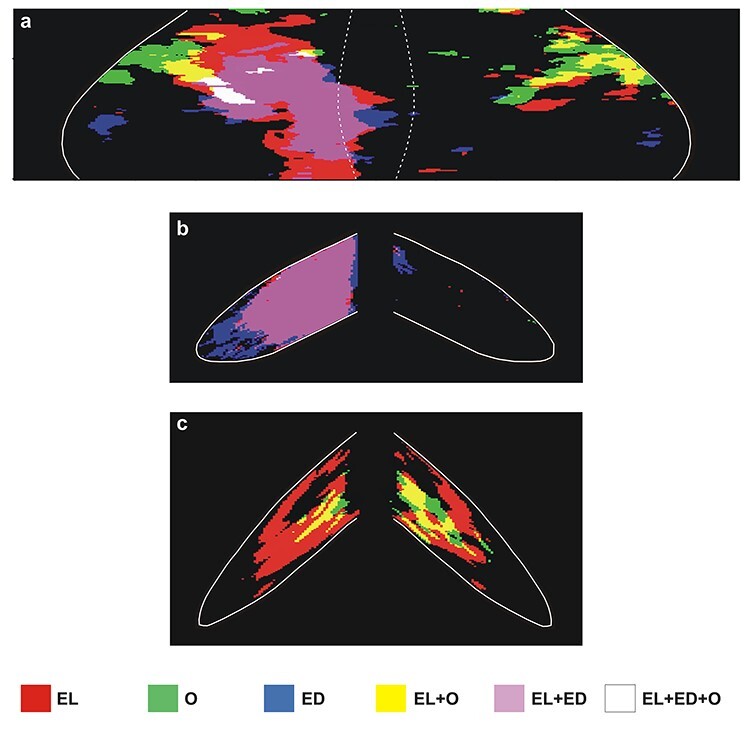
Superimposition of effects induced by 1) action execution in the light, 2) action execution in the dark, and 3) action observation. Superimposed activations in the vermian lobules IV-VI and their hemispheric extension (*a*), the hemispheric extension of the vermian pyramidal lobule VIIIB (*b*), and the Crus IIp of the ansiform lobule (*c*). Net effects higher than 10% are color coded red, green, or blue to represent the activations induced by action execution in the light, action observation, and action execution in the dark, respectively. Yellow stands for activations induced by both execution in the light and observation; violet indicates activations induced by both execution in the light and in the dark; white represents the overlap among all 3 conditions.

In Figure 13, only the spatial distribution and not the intensity of activations is represented. Figure 13*a* illustrates the spatial relationship of the significantly activated regions in the anterior and simple lobules of cerebellum 1) for action execution in the light in red, 2) for action observation in green, and 3) for action execution in the dark in blue. In this panel, the region of overlapping activations (red + blue = violet) demonstrates that a large portion of the neural space in the paravermal and a smaller part in the lateral cerebellar extension of lobules V/VI is activated in common for execution in the light and in the dark. Also, yellow represents the region of overlap between execution in the light and observation (red + green = yellow), and white represents the overlap among all 3 conditions (red + green + blue = white). Apparently, execution in the light (in red) covers the biggest neural space in the hemisphere ipsilateral to the moving forelimb, while observation (green) extends more laterally in both cerebellar hemispheres. Figure 13*b* illustrates the spatial relationship of the significantly activated regions in the hemispheric extension of pyramis lobule VIIIΒ of the cerebellum for action execution in the light (red) and in the dark (blue). Apparently, only action execution (both in light and in dark) activated the hemispheric extension of lobule VIIIΒ ipsilateral to the moving forelimb, whereas no activation was induced by action observation. Figure 13*c* illustrates the spatial relationship of the significantly activated regions in Crus IIp of the ansiform lobule, extension of vermian lobule VIIB. Apparently, Crus IIp was bilaterally activated for execution in the light and action observation, while it remained unaffected by action execution in the dark.

## Discussion

### General Considerations

The cerebellum was originally suggested to operate as a control center for preprogramming volitional limb movements (Ito 1970; Evarts 1975). More recently, a topographic organization of the human cerebellum was suggested, with the anterior lobe and lobule VIII representing the sensorimotor cerebellum, and with lobules VI and VII of the posterior lobe including the cognitive cerebellum (Stoodley and Schmahmann 2010). Here, we demonstrate that the primate cerebellum is involved in observation of actions performed by other subjects. Using the quantitative ^14^C-DG method, we revealed the spatial distribution of effects and the intensity of activations in the cerebellum induced by action execution in the light and in the dark as well as by action observation. Our results provide strong evidence that specific regions of the cerebellum are involved not only in the execution of a reaching/grasping action but also in the observation of the same action performed by another subject. We discuss these cerebellar findings and we examine them in the light of previously reported findings in the same monkeys, within cerebral frontal premotor and motor areas (Raos et al. 2004, 2007), parietotemporal somatosensory and temporo-occipital visual cortical regions (Evangeliou et al. 2009; Kilintari et al. 2011, 2014), prefrontal and occipito-parieto-temporal association areas (Raos and Savaki 2016, 2017), as well as within the spinal cord (Stamos et al. 2010).

### Effects Induced by Action Execution

Traditional forelimb representations in the cerebellum include one within lobules V/VI and another one in lobule VIII. Already 7 decades ago, experiments involving proprioceptive, tactile, auditory, and visual stimulation in monkeys demonstrated 2 simiusculi: one upside down located in the superior (anterior) and a second one upside up in the inferior (posterior) cerebellum (Snider and Eldred 1952). In our study, we found both these forelimb representations, that is, one in lobules V/VI and another one in lobule VIII, activated for action execution ipsilateral to the moving forelimb. However, we found 2 activated regions within lobules V/VI (see Fig. 5EL-CL). One activated region was located in the paravermal zone and another one more lateral in the hemispheric extension of lobules V/VI. These 2 regions may correspond to the 2 zones described in monkeys to be connected with the contralateral primary somatosensory–motor cortex, zones C2 and D2. Explicitly, the hemispheric extension of lobules IV/V/VI has been subdivided into the zones C2, D1, D2 in monkeys, from medially to laterally (Voogd 2014). The zones connected with the forelimb representations in the primary somatosensory–motor cortex were C2 and D2, separated by the zone D1 in between them, which was not connected with these cortices (Voogd 2014). Furthermore, these 2 activated regions of lobules V/VI in our study are reminiscent of the 2 hand representations recently demonstrated in these lobules of the human cerebellum by an fMRI study (Schlerf et al. 2010). In the latter report, a previously undiscovered somatotopic organization in human neocerebellar lobules VI/VII was demonstrated to be adjacent to that traditionally described in lobules IV/V/VI.

Our finding that the paravermal zone of lobules V/VI is activated ipsilateral to the moving forelimb for action execution, both in the light and in the dark (Figs 5 and 6), is compatible with previous reports. Neuroanatomical and neurophysiological studies have shown that the paravermal zone of lobules V and VI contains a representation of the forelimb (Adrian 1943; Snider and Eldred 1952; Sasaki et al. 1977; Jasmin and Courville 1987). More specifically, it was reported that neuronal activity in the paravermal portions of lobules V and VI is related to reaching movements with the forelimb (Thach 1970; Mano and Yamamoto 1980; Fortier et al. 1989). The significant activation of the paravermal zone of lobules V/VI, which we found in the present study, may have been triggered by inputs from the cerebral sensorimotor cortex via the corticopontine and pontocerebellar pathways, and from the moving limb via the spinocerebellar and cuneocerebellar tracts (Adrian 1943; Evarts and Thach 1969; Voogd et al. 1969; Jasmin and Courville 1987). Indeed, the activation of the paravermal zone of lobules V/VI ipsilateral to the moving forelimb, reported here, is linked to 1) the activation of the forelimb representations in the contralateral primary motor and somatosensory cortices [see Table 1 in (Raos et al. 2004)] and 2) the activation of the ipsilateral spinal forelimb representation [Table 1 in (Stamos et al. 2010)], activations which we have found in previous studies in the same monkeys during reaching/grasping movements. With this dual input, the paravermal regions of the anterior and simplex lobes (containing lobules V and VI, respectively) may compare information about intended movement from the contralateral cerebral cortex with information about actual movement from the ipsilateral limb, in order to correct for mismatches between the 2 (Oscarsson 1979; Stein 1986). The above proposals regarding the paravermal zones of lobules V and VI may also apply to the paravermal zone of lobule VIII. This region, which was also activated ipsilateral to the moving forelimb during action execution both in the light and in the dark (Figs 7 and 8), receives cerebrocerebellar input via the pontine nuclei (Brodal and Bjaalie 1992), as well as spinocerebellar input (Voogd et al. 1969; Ekerot and Larson 1979), and olivocerebellar afferents (Groenewegen et al. 1979; Brodal and Kawamura 1980). Interestingly, during action execution in the dark, the paravermal regions of lobules V/VI and VIII displayed stronger activation than during execution in the light (see Tables 1 and 3). This finding may reflect increased input from the somatosensory cortex to the cerebellum in order to compensate for the lack of visual input during action execution in the dark (Savaki et al. 1996). Moreover, our finding that the activation in the lateral hemispheric extension of the lobules V/VI and VIII was stronger for execution in the light than in the dark (see Tables 1 and 3) agrees well with previous reports that the lateral cerebellar zone is more closely related to cognitive functions, whereas the paravermal zone is related mainly to forelimb sensorimotor functions (Brodal 1979; May and Andersen 1986; Schmahmann 2019; Schmahmann et al. 2019).

In the ansiform lobule, which is considered as the main nonmotor area of the cerebellum, Crus IIa remained unaffected, whereas Crus IIp was activated in our study. In more detail, Crus IIa, which is reciprocally connected with the prefrontal cortical area 46 (Kelly and Strick 2003), was affected by neither action execution nor action observation (see Figs 8 and 9). In contrast, Crus IIp, which is predominantly interconnected with the posterior intraparietal area MIP (Prevosto et al. 2010) was activated for execution in the light and action observation but not for execution in the dark (see Figs 9 and 10). The latter finding, in association with our previous report that area MIP was activated by both execution in the light and observation of grasping/reaching movements in the same monkeys, indicates that the Crus IIp-MIP loop supports both the execution and the perception of visually guided arm movements. Indeed, MIP is an arm movement-related area, involved in sensory guidance of reaching (Mountcastle et al. 1975; Kalaska and Crammond 1992; Evangeliou et al. 2009), containing bimodal neurons with congruent visual–somatosensory receptive fields. Apparently, the Crus IIp-MIP loop forms a suitable neural substrate not only for online control of movements but also for predictive control of actions. Most probably, this loop allows not only for correction of movement during execution but also for perception of observed actions, based on an efference copy of motor signals and visual and proprioceptive feedback in both cases (Blakemore and Sirigu 2003).

### Effects Induced by Action Observation

Observation of reaching/grasping movements performed by another subject induced significant activation in the observer’s cerebellum, only in a restricted region of the lateral-most hemispheric extension of lobules V/VI, while lobule VIII remained unaffected (see Figs 5 and 7). The lack of activation in the paravermal lobules V/VI and in lobule VIII for action observation in our study may be associated with the metabolic suppression of the forelimb representation in the spinal cord, which was found exclusively for action observation and not for action execution (Stamos et al. 2010). This suppression of spinal activity may deprive the paravermal lobules V/VI and the lobule VIII from an excitatory input.

Our finding that the paravermal cerebellum was not activated whereas Crus IIp was activated by action observation agrees well with the report that whereas lesions of the anterior and posterior medial sensorimotor cerebellum lead to the cerebellar motor syndrome of ataxia and dysmetria, lesions of the posterior cognitive cerebellum (including Crus IIp) produce dysmetria of thought (Schmahmann 2019; Schmahmann et al. 2019). It also agrees well with 2 meta-analyses of the human functional imaging literature, which have demonstrated that cerebellar activation patterns are task dependent, with broad sensorimotor (anterior lobe, medial lobule VI, lobule VIII) and nonmotor (lateral posterior hemispheres, including lateral lobule VI and crus II) (Stoodley and Schmahmann 2009; Keren-Happuch et al. 2014) regions activated separately. Also our finding that the lateral-most zone of lobules V/VI as well as Crus IIp is activated for action observation is compatible with the report that cerebellar lesions affecting Crus II induce cognitive impairment (Stoodley and Schmahmann 2010). Finally, our finding that the cerebellum is engaged in action observation is compatible with previous brain imaging studies in humans as well as nonprimate lesion data suggesting that the cerebellum is involved not only in movement coordination but also in visual action observation (Leggio et al. 2000; Gallagher and Frith 2004; Calvo-Merino et al. 2006; Frey and Gerry 2006; Gazzola and Keysers 2009; Abdelgabar et al. 2019). More specifically, our finding that action observation activates the lateral part of cerebellar hemispheres is in agreement with fMRI studies reporting that the lateral cerebellum is selectively activated during observation of biological motion (Vaina et al. 2001; Sokolov et al. 2010) and during observation of manipulative actions (Errante and Fogassi 2020). Indeed, it has been reported that the output neurons of the lateral cerebellum are related to more general aspects of the task (Robertson and Grimm 1975), such as the sensory inputs guiding movements (Spidalieri et al. 1983; Marple-Horvat and Stein 1990) and the “expectation” or motor set (Grimm and Rushmer 1974; Strick 1983). Our findings also complement the report that damage to the lateral cerebellum causes a pronounced deficit in visual perception of human locomotion, whereas medial lesions do not substantially affect perception of human walking (Sokolov et al. 2010). Interestingly, it was proposed that, in addition to the prediction apparatus, the lateral cerebellum contains apparatus for generating a plastic internal representation of the sensory–motor model of actions that could be used to predict, rehearse, and optimize the performance of a subject (Stein 1986). Such a sensory–motor model of actions could participate in action perception, explaining the lateral cerebellar activation for action observation in our study.

### Concluding Remarks

In summary, given the corticocerebellar networks activated in the present and our previous studies in the same monkeys, it is likely that on one hand the more lateral hemispheric extensions of the cerebellar lobules V/VI and VIII, as well as Crus IIp of the ansiform lobule, receive visual information about target and arm position from the occipitoparietal and middle temporal cerebral cortex. Therefore, the lateral hemispheric extension of the cerebellum is activated during both execution and observation in the present study. On the other hand, the paravermal extensions of lobules V/VI as well as lobule VIII receive information about the intended limb movement from the contralateral somatosensory–motor cerebral cortex, and about the moving forelimb from the ipsilateral spinal cord, only during execution. Therefore, the paravermal cerebellum is activated only for execution in our study. These visual and somatosensory–motor inputs to the cerebellum along with the cerebellar outputs to the cerebral cortex may be repeatedly updated and adjusted to coordinate (during execution) or to participate in understanding of (during observation) the forelimb movements. Finally, activation of the lateral-most hemispheric extension for action observation in our study supports the suggestion that, while the paravermal cerebellum helps to execute voluntary limb movements and the lateral hemispheric extension is involved in visual guidance of the movement, the extreme lateral (lateral-most) cerebellar hemispheres are involved in more cognitive functions such as action perception. In other words, we suggest that there may be a gradient of action representation in the cerebellum. Motor aspects of actions may be represented mainly medially, visuomotor aspects more laterally, and cognitive aspects in the extreme lateral hemispheres. There may be a gradual organization of modularity that progresses from unimodal (medially) to multimodal (laterally) functional areas of the primate cerebellum. This suggestion is in agreement with a recent report describing a principal gradient of the macroscale function in human cerebellum. It was reported that functional areas in the human cerebellum follow a gradual organization progressing from primary (motor) to transmodal (task-unfocused) regions (Guell et al. 2018), following the principle of organization in the cerebral cortex, that is, the gradual progression away from areas processing unimodal information (motor/somatosensory, auditory, and visual primary cortices) to areas processing more abstract, transmodal information. In other words, our study supports the existence of a sensorimotor to cognition modularity gradient in the primate cerebellum.

## Funding

“Advanced Research Activities in Biomedical and Agroalimentary Technologies” (MIS 5002469), which was implemented under the “Action for the Strategic Development on the Research and Technological Sector,” funded by the Operational Programme “Competitiveness, Entrepreneurship, and Innovation” (NSRF 2014-2020), and cofinanced by Greece and the European Union (European Regional Development Fund). Funding source had no involvement in study design; in the collection, analysis, and interpretation of data; in the writing of the report; and in the decision to submit the article for publication.

## Notes

We thank S. Bakola, M. Evangeliou, G.G. Gregoriou, M. Kilintari, and A. Stamos for participating in the experiments and A. Stamos and M. Kefalogianni for help with autoradiographic imaging. *Conflicts of Interest:* None declared.
